# Miliary tuberculosis presenting as bilateral pseudo-retinoblastoma

**DOI:** 10.3205/oc000185

**Published:** 2021-07-28

**Authors:** Yamini Attiku, Pukhraj Rishi

**Affiliations:** 1 Shri Bhagwan Mahavir Vitreoretinal Services, Sankara Nethralaya, Chennai, Tamil Nadu, India

**Keywords:** eye, retinoblastoma, tuberculosis

## Abstract

**Objective:** To describe an interesting case of miliary tuberculosis mimicking retinoblastoma.

**Method:** A retrospective case report.

**Result:** The twin brother of a known case of retinobastoma presented with headache. On fundus examination, multiple yellowish-white lesions were noted in both eyes. Magnetic resonance imaging of the brain showed multiple enhancing lesions. A diagnosis of miliary tuberculosis was made and anti-tubercular therapy was started.

**Conclusion:** Ocular tuberculosis can mimic retinoblastoma and lead to diagnostic dilemma especially in cases with family history of retinoblastoma.

## Introduction

Retinoblastoma is the most common childhood intraocular malignancy. Several pediatric ocular conditions can have similar presentation. The common causes of pseudoretinoblastoma include Coats disease, persistent fetal vasculature, familial exudative vitreoretinopathy, vitreous hemorrhage, toxocariasis, retinal detachment, coloboma, astrocytic hamartoma and endogenous endophthalmitis [1], [2]. These conditions can be potentially misdiagnosed as retinoblastoma which can lead to inappropriate treatment with focal therapy, systemic chemotherapy, intra-arterial and intra-vitreal chemotherapy or even enucleation. Hence, early recognition, accurate diagnosis and appropriate treatment are necessary.

## Case description

**Figure 1:**
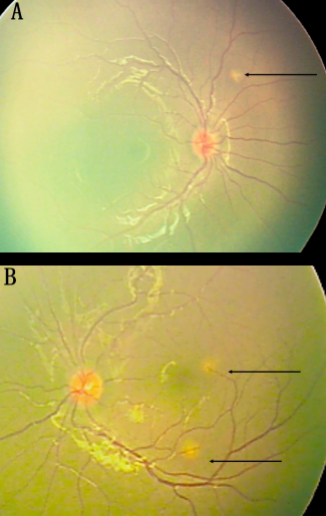
Fundus photo of the right and left eye showing choroidal tubercles in both the eyes

**Figure 2:**
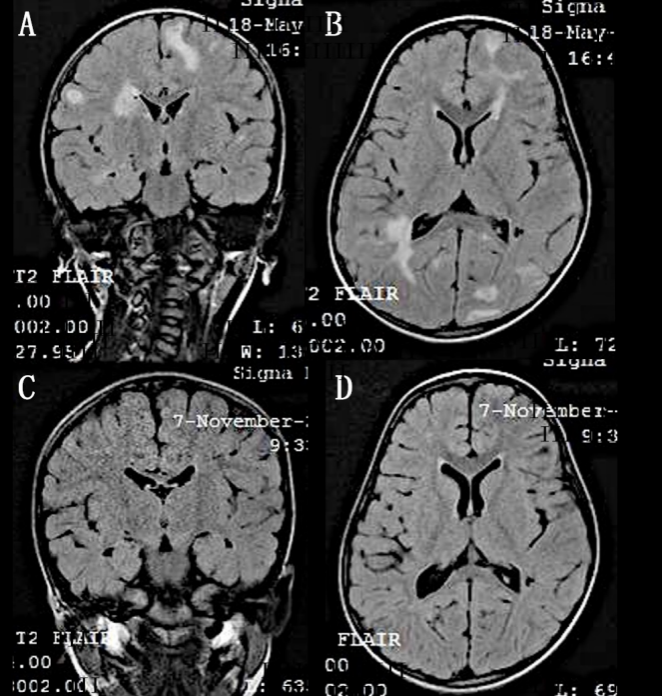
A, B) MRI brain showing enhancing lesions with perilesional edema in the supratentorial and infratentorial brain parenchyma. C, D) These lesions disappeared following treatment.

**Figure 3:**
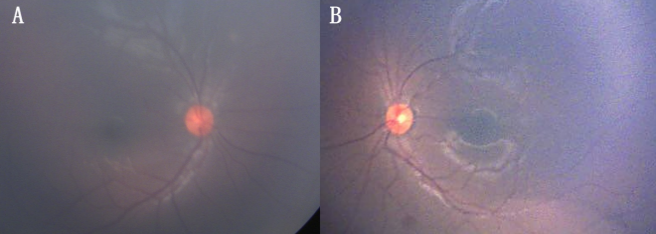
Fundus photo of the right and left eye showing resolution of lesions in both the eyes following treatment with anti-tubercular therapy

The dizygotic twin brother of a known case of bilateral retinoblastoma, born following in-vitro fertilization was being screened annually for retinoblastoma. There was no other family history of retinoblastoma. No pathology was noted in the first two years of screening. At 3 years of age, he presented with headache. Vision was 20/20 in both eyes. Examination under anaesthesia revealed multiple yellowish-white retino-choroidal lesions. A solitary lesion of size 0.5 mm in the right eye superior to the disc and two similar lesions in the left eye, one superior to fovea and the other inferior to the disc (Figure 1A, B (Fig. 1)). Magnetic resonance imaging (MRI) brain revealed multiple enhancing lesions (Figure 2A, B (Fig. 2)). Pediatrician and neurologist opinion was sought for, who investigated the child for tuberculosis. Tuberculin skin test was 20 mm and QuantiFERON TB gold test was positive. The diagnosis of miliary tuberculosis was made. The patient was treated with anti-tubercular treatment for 6 months which resulted in resolution of lesions (Figure 3A, B (Fig. 3)). The CNS lesions too resolved following treatment (Figure 2C, D (Fig. 2)).

## Discussion

Choroidal tuberculoma can mimic retinoblastoma, especially in children with a strong family history of retinoblastoma. Among the various mimickers of retinoblastoma, choroidal tubercle is a rare differential diagnosis. Few cases of ocular tuberculosis have been misdiagnosed as retinoblastoma and have undergone enucleation, only to be diagnosed as tuberculosis later on histopathology [3], [4], [5]. Appropriate diagnostic imaging and laboratory investigations help in making an accurate diagnosis. In comparison to tuberculin skin test, QuantiFERON TB gold test is reported to be less influenced by previous BCG vaccination in diagnosing active tuberculosis infection in children [6].

## Conclusion

In a child with systemic tuberculosis presenting with retinochoroidal lesions, ocular tuberculosis should be strongly suspected. Rarely choroidal tubercles can mimic retinoblastoma and lead to diagnostic dilemma especially in children with family history of retinoblastoma.

## Notes

### Competing interests

The authors declare that they have no competing interests.
